# Youth Soccer Heading Exposure and Its Effects on Clinical Outcome Measures †

**DOI:** 10.3390/sports12120342

**Published:** 2024-12-10

**Authors:** Victoria E. Wahlquist, Thomas A. Buckley, Jaclyn B. Caccese, Joseph J. Glutting, Todd D. Royer, Thomas W. Kaminski

**Affiliations:** 1 Department of Kinesiology and Applied Physiology, University of Delaware, Newark, DE 19716, USA; vwahlq@udel.edu (V.E.W.); tbuckley@udel.edu (T.A.B.); royer@udel.edu (T.D.R.); 2 School of Health and Rehabilitation Sciences, The Ohio State University College of Medicine, Columbus, OH 43210, USA; jaclyn.caccese@osumc.edu; 3 School of Education, University of Delaware, Newark, DE 19716, USA; glutting@udel.edu

**Keywords:** adolescent, repetitive head impacts, purposeful heading, brain health, physical activity, balance, neurocognition

## Abstract

Purposeful heading, in which players may use their heads to advance the ball in play, is a unique part of soccer. Clinical outcome measures used to aid in the diagnosis of a concussion have long been a cornerstone of the contemporary measurements associated with the short- and long-term effects of monitoring repetitive head impacts (RHI) and soccer heading exposure. The effects of RHI in the youth population are still unknown, therefore, the purpose of this study was to examine if heading exposure is predictive of changes in self-reported symptoms, neurocognitive functioning, gait, and balance in female youth soccer players over the course of one soccer season. Small improvements in neurocognitive functioning and gait and slight deficits in balance were observed from pre- to post-season. All changes were not clinically relevant and likely due to a practice effect. The low heading exposure in our cohort of youth soccer players was likely not enough to elicit any changes in clinical measures. In general, our clinical outcomes did not change after a season of soccer play and change scores were not predicted by heading exposure.

## 1. Introduction

Purposeful heading, in which players may use their heads to advance the ball in play, is a unique part of soccer. This skill is typically taught after youth players have learned and practiced the fundamentals of dribbling, passing, shooting, etc …. In the United States and several other countries, guidelines on soccer heading in youth soccer players are in place to limit early exposure to repetitive head impacts (RHI). For example, the guidelines set forth by the United States Soccer Federation (US Soccer) prohibit heading for children 10 years old and younger, while those aged 11–13 are restricted by weekly practice exposure limits, however in game heading is unrestricted. Like most skills, purposeful heading in soccer takes practice to properly execute and master the technique. Purposeful soccer headers have the potential to produce RHI similar to tackles in American football and body checks in ice hockey. The effects of RHI are still unknown, however, there is speculation that long-term detrimental effects on neurocognitive functioning may occur due to higher levels of RHI. Contrasting results have been reported in soccer involving neurocognitive function whereby some have reported deficits in relation to RHI while others have not [1,2,3,4,5]. Other clinical outcome measures such as balance and concussion-related symptoms have also been commonly studied in relation to RHI [6,7,8,9]. Researchers speculate that RHI can lead to athletes developing chronic traumatic encephalopathy (CTE), a heavily discussed topic in American football [10,11,12]. Much of the current CTE research is limited to small, biased populations and do not yet have conclusive evidence on the relationship between RHI and CTE [3,13]. Research on CTE which includes soccer athletes is very limited at this time [14,15,16].

Clinical outcome measures used to aid in the diagnosis of a concussion have long been a cornerstone of the contemporary measurements associated with the short- and long-term effects of monitoring RHI and heading exposure. Self-reported symptoms are one of the common clinical outcome measures used in concussion diagnosis and research. The most common symptom reported after a concussion by all ages is headache; additionally many report dizziness and difficulty concentrating [17,18,19,20]. Females generally tend to report more symptoms than males after sustaining a concussion [21,22,23] Self-reported symptoms have been reported to increase after a bout of soccer heading, with 50% of youth reporting a headache immediately afterwards [6,9,24,25]. Interestingly, when symptoms were observed from pre- to post-season there was no change, which may suggest little to no effect from heading exposure [7,26]. Having a useful and pertinent symptom monitoring system available for use in the youth athlete population is important from both a medical and research standpoint. The symptom checklist on the Child Sport Concussion Assessment Tool 5th Edition (Child SCAT 5) has been adjusted to be scored on a 4-point Likert scale, with symptoms written in age-appropriate terms while also being validated in the youth population [27,28,29].

Another common clinical outcome measure is neurocognitive function. Typically, athletes who sustain a concussion experience a decrease in cognitive functioning [22,23,30]. Females have also been reported to have a greater decline in neurocognitive functioning than males [22,23]. The utilization of neurocognitive functioning in assessing the effects of heading exposure has led to varying results. Several studies observed no changes from pre- to post-season, after a bout of heading, or over the course of a high school career [7,26,31,32,33,34]. However, other studies have reported neurocognitive impairment after an acute bout of heading, comparing soccer players to non-soccer players, and related to heading exposure over a period of time [35,36,37,38,39,40]. Aspects of neurocognitive functioning that are commonly tested include, but are not limited to, information processing speed, attention, memory function, and executive function. Information processing speed can be measured using a simple reaction time test and tests a person’s ability to quickly process information and react to a stimuli [41,42]. The Child SCAT5 also includes cognitive tests that assess attention and memory function and have been adjusted to be age-appropriate for youth [28,29,43]. Executive function occurs in the frontal lobe of the brain and controls mental skills such as working memory, flexible thinking, and self-control, which all play a role in everyday tasks [39,44].

Balance is also commonly utilized in concussion diagnosis. Following a concussion, athletes will display a disruption in their balance [45,46,47,48]. The use of balance in determining the effects of soccer heading exposure has resulted in contrasting outcomes. Some studies reported a change in all age groups in postural control and higher sway velocity following a bout of soccer heading [8,49]. Other studies have found no changes in balance after a bout of heading or from pre- to post-season [6,32,50,51]. Very few of these studies were conducted in the youth population [8,45]. The advancement in technology has allowed various balance tests to now be completed with portable devices such as a smartphone or computer tablet. The SWAY Balance System utilizes an accelerometer in the smartphone to measure thoracic sway and has been reported to be comparable to other balance tests [52,53,54]. Another test of balance and postural control is the tandem gait test. It is performed as a single-task and a dual-task, which adds a cognitive task while performing the heel-to-toe walking. The tandem gait test evaluates dynamic balance, speed, and coordination and the execution of motor and cognitive demands simultaneously [55,56]. This test is a reliable tool for the youth population and concussed athletes have been reported to demonstrate slower times than a healthy population [56,57,58,59]. It has been observed that the tandem gait test is more sensitive than other common static balance tests and the added dual-task component has demonstrated deficits even after the single-task score returned to normal [59,60]

The youth population is of particular interest for RHI and studying common clinical outcome measures. The youth are a vulnerable population in relation to brain function since they are still developing both physically and mentally. As it pertains to the 2015 US Soccer Heading Guidelines, those soccer players aged 11–13 years are also allowed to purposefully head the ball in practice and games which causes concern for parents and the potential effects this may have on their mental development. While it is understood that female soccer players experience a higher number of concussions, it was recently reported that female players tend to close their eyes and fail to use better defensive posturing during aerial challenges when compared to their male counterparts [61]. Such differences likely contribute to the higher incidence rates of concussion in female soccer players. The effects of RHI in the youth population are still unknown, therefore, the purpose of this study was to examine if heading exposure predicts changes in self-reported symptoms, neurocognitive functioning, and balance in female youth soccer players over the course of one soccer season. We hypothesized that heading exposure would predict an increased change in self-reported symptoms; and that as heading exposure increased, neurocognitive functioning and balance would worsen.

## 2. Materials and Methods

### 2.1. Participants

Sixty-one female youth soccer players were recruited from two local soccer clubs (Table 1). Participants played on competitive travel soccer teams in the U12 (19 players), U13 (28 players), and U14 (14 players) age groups. The study was conducted according to the guidelines of the Declaration of Helsinki, and approved by the Institutional Review Board of the University of Delaware (UDIRB 1168008-7). All participants signed informed assent forms and parents/guardians signed informed parental permission forms. Participants were excluded from the study if they were injured (orthopedic or concussion) at the time of testing or had a neurological disorder that would prevent them from completing the clinical outcome assessments. Participants completed concussion assessment tests pre- and post-season. The soccer seasons were 8–10 weeks in duration and held in either the Fall or Spring.

### 2.2. Instrumentation

Heading exposure was captured through video recording with an AKASO Brave 6 action camera (AKASO, Frederick, MD, USA). Video recordings were saved directly to an SD memory card for easy transfer to a computer for a detailed analysis of the actual number of headers. Static balance was measured using two devices: the SWAY Balance System (SWAY Medical, Tulsa, OK, USA) and the Tekscan MobileMat (Tekscan, Norwood, MA, USA). The SWAY Balance System can be downloaded to any Apple or Android operating systems and utilizes the built-in accelerometer of the device to measure thoracic sway. The Tekscan MobileMat was connected to a laptop and utilized through the FootMat Research (Tekscan, Norwood, MA, USA) application software to capture center of pressure (COP). Sway velocity, 95% confidence ellipse area, and sway frequency, Ref. [62] using raw COP data from the Tekscan MobileMat, were calculated using an in-house Labview (National Instruments, Austin, TX, USA) program. A phone stopwatch function was used to capture the time for the tandem gait and Trail Making Test A and B.

### 2.3. Procedures

#### 2.3.1. Demographic Data

Participants completed a general health and concussion history questionnaire (Figure S1) containing general information including age, height, mass, sex, playing position, any previous injuries within the last 6 months, and concussion history. A member of the research team led each participant through the general health and concussion history questionnaire to ensure every part was completed. If a participant did not know the answer to a question, a parent was asked to help answer the question.

#### 2.3.2. Heading Exposure

**Figure 1:**
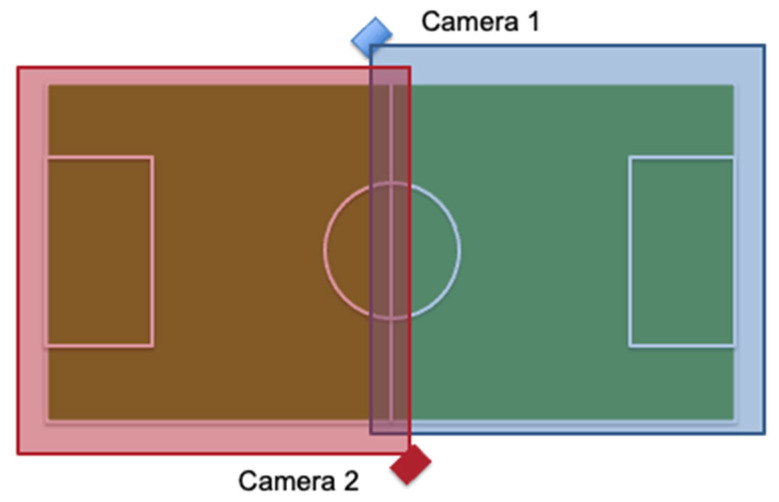
Game Camera Setup on Soccer Field.

Coaches conducted practices and games with their normal tactics and plans. All practices and games of each team for one soccer season were videotaped to record head impact exposure of each participant. The video camera(s) was set up at the side of the playing field on a tripod from a strategic vantage point that allowed for a recording of the entire soccer practice/game. Each team assisted in the videotaping of practices and games. Teams typically practiced on a smaller portion of the soccer field and the whole practice was captured with 1 video camera. For practices that used a larger portion of the soccer field, 2 video cameras were used to capture the practice. Games were captured with 2 video cameras setup, as seen in Figure 1, with each camera capturing half of the soccer field and a slight overlap at the middle of the field. Video was transferred to an external hard drive each week by the research team. Videos were viewed by the research team to record athlete exposures and count the number of headers performed by each participant. As this age group of female youth soccer players are still in the early heading skill acquisition phase, instances where other potential head impacts (falls, aerial challenges, etc. …) were rare and therefore not accounted for. In fact, during the entire data collection time period, there were no instances of sport-related concussion reported. Reliability for the video capture methodology was previously established [63].

#### 2.3.3. Self-Reported Symptoms

Participants completed a 21-item symptom checklist from the Child SCAT5 (Figure S2). Symptoms were rated on a 4-point Likert scale where 0 was “not at all/never” and 3 was “a lot/often”. The symptom checklist was completed according to how the participant was feeling at the moment they were completing the checklist. The total symptoms and total symptom severity were tallied up for a maximum total of 21 and 84 respectively.

#### 2.3.4. Static Balance

**Figure 2:**
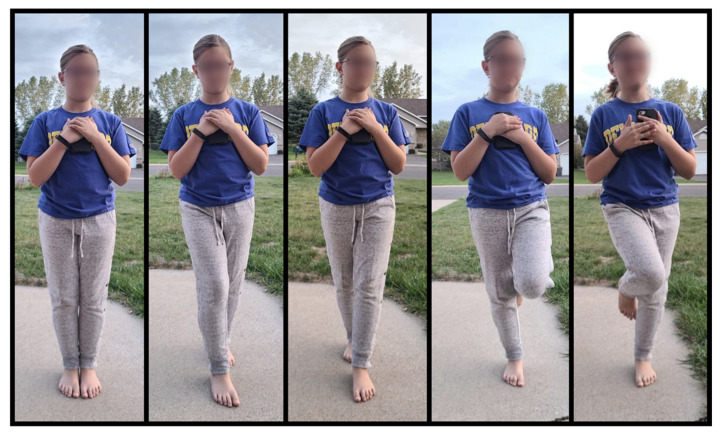
SWAY Stances (**left** to **right**) Both feet, tandem right foot in front, tandem left foot in front, single leg right, and single leg left.

Static balance was first measured utilizing the Sports protocol in the SWAY Balance System. The protocol involved 5 different stance positions (feet together, tandem with left foot in front, tandem with right foot in front, single leg on the right foot, and single leg on the left foot) and had on-screen instructions for the participant to follow (Figure 2). Each stance test was completed with the participant’s eyes closed and lasted for 10-s. Participants were instructed that they could put their foot down if they felt off-balance or if they were going to fall, but were instructed to resume the test position as quickly as possible. SWAY balance was assessed with our participants barefoot. Each stance was a percentage of 100 with 100% being completely still with no sway.

The second method utilized to measure static balance was the Tekscan MobileMat. Data was collected at 100 Hz for 20 s. COP data was captured for 3 stances: feet together, single leg on the right foot, and single leg on the left foot. Participants completed 1 trial for each stance with each trial lasting 20 s. Participants were instructed to close their eyes and have their hands on their hips for each trial. Participants who lost their balance were instructed to resume the test position as quickly as possible.

#### 2.3.5. Reaction Time

**Figure 3:**
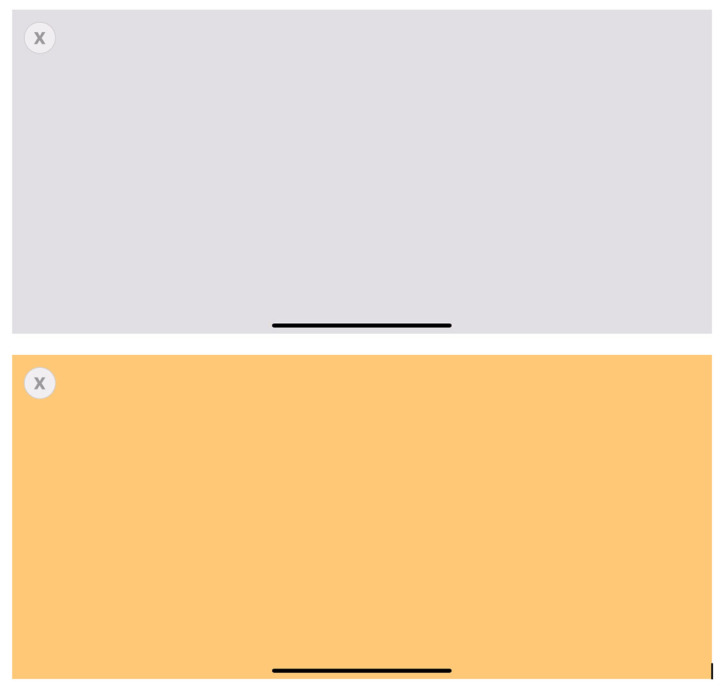
SWAY Reaction Time White and Orange Screen.

Reaction time was an additional task from the Sports protocol in the SWAY Balance System that we utilized. Participants held the smartphone with both hands in front of them. Once the screen turned from white to orange (Figure 3), they quickly shook the phone in any direction. The time that it took for the participant respond to the stimulus was recorded as the reaction time. It included 5 trials with the fastest and slowest trials dropped while the mean of the remaining 3 trials was used for the final outcome measure.

#### 2.3.6. Cognitive Function

The Step 3: Cognitive Screening and Step 5: Delayed Recall of the Child SCAT5 (Figure S3) were utilized to test cognitive functioning. Specifically, immediate memory, concentration, and delayed recall were tested. For immediate memory, 5 words were read by the researcher and the participant then repeated back as many words as they could remember in any order. Three trials were completed with the same words. Each correct word counted as 1 point for a maximum of 5 points per trial and 15 points total. For the concentration segment, it included the researcher reading a string of numbers and the participant repeating the numbers in reverse order. If the participant repeated back the string correctly, they moved on to the next string which included one more number (total of 5 different string lengths starting at two numbers going up to six numbers). If the string was repeated incorrectly, the participant had one more chance at that string length. Each correct string of numbers was worth 1 point for a maximum total of 5 points. Concentration also included reciting the days of the week in reverse order starting with Sunday. The participant received 1 point if they correctly listed the days of the week in reverse order. The total concentration score was the number string and days of the week scores added together for a total of 6 points. For delayed recall, the participant listed as many words as they could remember from the immediate memory words completed earlier for a maximum of 5 points.

The Trail Making Test Part A (TMTA) and B (TMTB) pen and paper version was utilized to test executive function (Figure S4). TMTA consists of 25 circles distributed over a piece of paper with the numbers 1–25 written in the circles. Participants were instructed to draw lines to connect the circles numerically from 1–25. TMTB included 24 circles with half of the circles containing the numbers 1–12 and the other half containing the letters A–L. The participants were instructed to connect the circles alternating from number to letter in order, 1-A-2-B, until they reached the final letter, L. Participants were timed, in seconds, on how long it took them to complete each test. The Trail Making Tests were completed after the days of the week and before the delayed recall of the Child SCAT5.

#### 2.3.7. Tandem Gait

**Figure 4:**
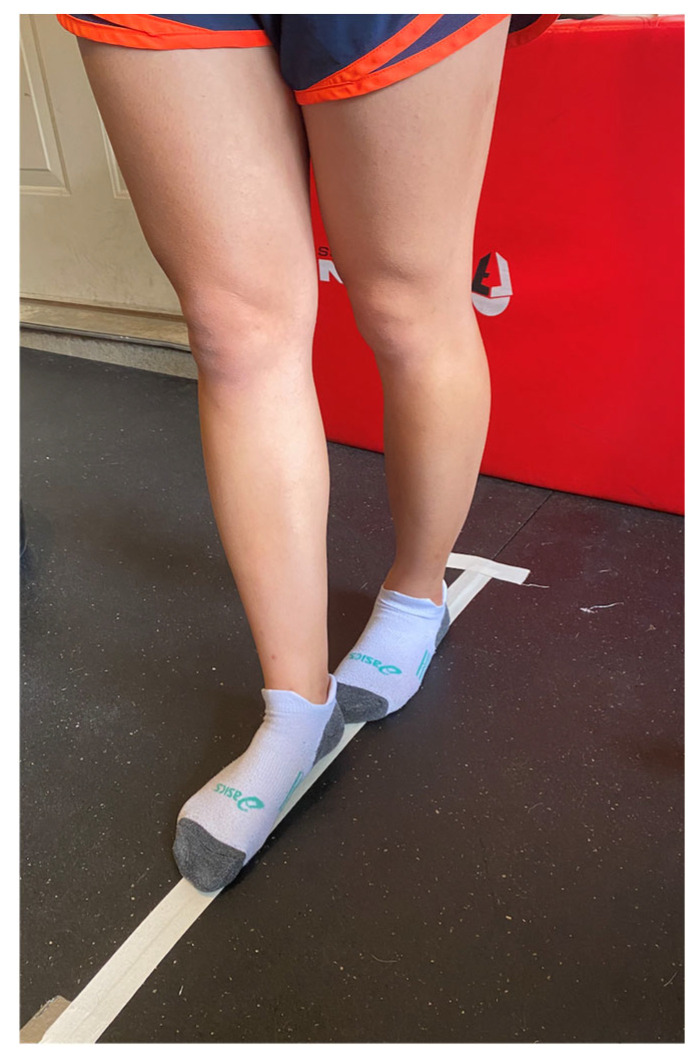
Tandem Gait Heel-to-Toe Pattern.

The tandem gait test was completed using a 3-m line with shoes removed and participants walking in a heel-to-toe gait pattern (Figure 4). Participants completed 3 timed trials each of the single-task and dual-task tandem gait protocol. Recent research suggests that less trials, as compared to 5 trials, can distinguish between healthy and concussed athletes [64]. In the single-task tandem gait protocol, participants started just behind the start line, walked the length of the line in the heel-to-toe gait pattern to the other end of the line, made a 180° turn, and walked back in the same manner to the start line. The time for each trial was recorded with the fastest time used for data analysis. Participants then completed 3 cognitive tasks while standing quietly: one 30 s trial for each cognitive task. The 3 cognitive tasks were: (1) spell a 5-letter word backwards, (2) subtract by 6 s or 7 s from a randomly assigned 2-digit number, and (3) recite the months in reverse order from a randomly assigned month. The total number of items attempted and number of items correct/incorrect were recorded. The dual-task tandem gait protocol was the same as the single-task but also included a cognitive task done simultaneously. One tandem gait trial for each cognitive task was completed. The time for each trial, total number of items attempted, and number of items correct/incorrect were recorded.

### 2.4. Statistical Analysis

COP data from the Tekscan MobileMat was trimmed if the COP was outside of the testing area resulting in a time less than 20 s (i.e., the participant stepped outside of the testing area). Sway velocity, 95% confidence ellipse area, and sway frequency from the COP data were calculated using an in-house Labview program. The equations for sway velocity, 95% confidence ellipse area, and sway frequency have been previously published [62]. Dual-task cost (DTC) was calculated for the tandem gait time with the following equation [65]:

$$DTC\left(\%\right)=\frac{\left(dual\text{-}task\;value\right)-(single\text{-}task\;value)}{single\text{-}task\;value}\times 100$$

Data were then analyzed using (1) dependent samples *t*-tests to compare measures from pre- to post-season and (2) linear regression analyses to determine if heading exposure predicted the change in symptom scores, neurocognitive functioning, and balance measures. The Benjamini-Hochberg correction was used to correct for multiple comparisons with the false discovery rate set at 0.25 [66,67]. Cohen’s *d* effect sizes were reported for significant findings in the dependent samples *t*-test to determine the standardized difference in the means. Cohen’s *f*^2^ effect sizes were reported for the significant findings in the linear regression analysis. An *a priori* alpha level of 0.05 was set to represent statistical significance. All data were analyzed using the IBM Statistical Package for the Social Sciences Version 28 (IBM SPSS Statistics, Armonk, NY, USA).

## 3. Results

Heading exposure for practices and games are shown in Table 2. Table 3 includes the means, standard deviations, and *p*-values for each outcome measure pre- and post-season. There were no statistically significant differences in self-reported symptom scores from pre- to post-season. Neurocognitive outcome measures that were statistically significant from pre- to post-season were immediate memory (*t* = −2.17, *df* [59], *p* = 0.034), concentration (*t* = −2.13, *df* [59], *p* = 0.038), TMTA (*t* = 3.51, *df* [59], *p* < 0.001), and TMTB (*t* = 2.83, *df* [58], *p* = 0.006). Immediate memory and concentration increased while TMTA and TMTB decreased which, for all of these variables, is an improvement from pre- to post-season. The single-task for the tandem gait test was statistically significant (*t* = 2.04, *df* [60], *p* = 0.046) with an improvement from pre- to post-season corresponding to a decrease in time. The dual-task cost for months in reverse was also statistically significance (*t* = 1.86, *df* [60], *p* = 0.067) with an improvement from pre- to post-season. The two balance measures that were statistically significant were sway velocity (*t* = −3.16, *df* [60], *p* = 0.002) and sway frequency (*t* = −2.48, *df* [60], *p* = 0.016) on the feet together stance. Sway velocity and frequency both increased over time corresponding to worse post-season scores. The reported effect sizes according to Cohen [68] are considered small and likely indicate a relatively small difference between the pre- and post-test measurements. Additionally, the TMTA and TMTB differences, along with the sway velocity are likely clinically meaningful, while the others suggest otherwise. All other comparisons of neurocognitive functioning and balance were not statistically significant.

**Figure 5:**
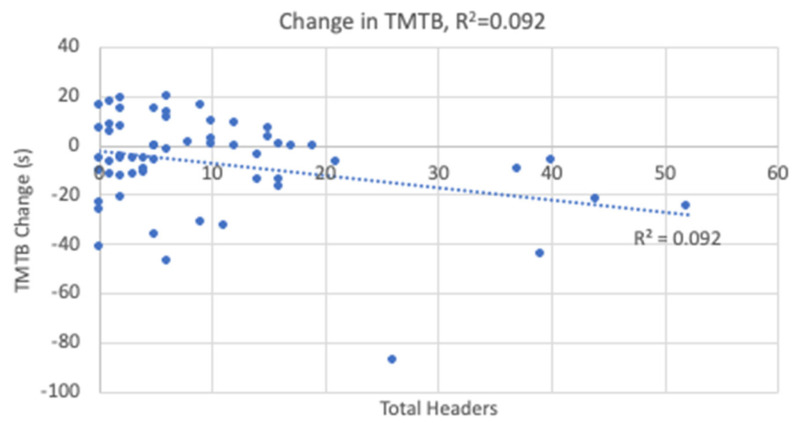
Change in Trail Making Test B Linear Regression.

**Figure 6:**
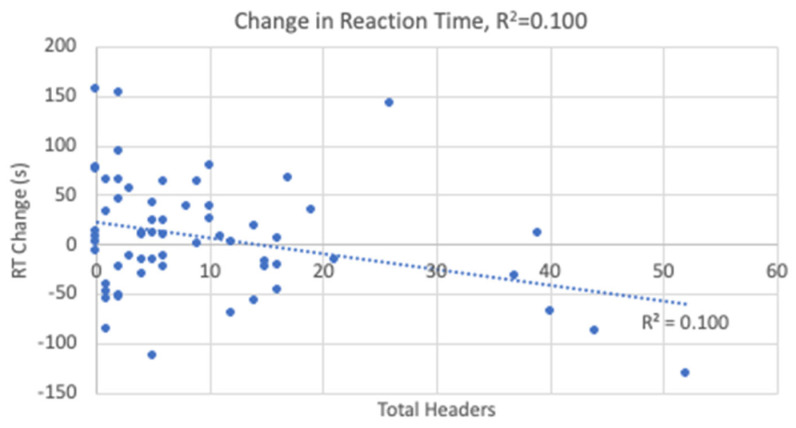
Change in Reaction Time Linear Regression.

The linear regression analyses resulted in statistical significance for the changes in TMTB and reaction time with the results shown in Table 4. Heading exposure predicted 9.2% of the variance in TMTB (Figure 5) and 10% of the variance in reaction time (Figure 6). Higher heading exposure for both TMTB and reaction time corresponded to significant improvements or decreases in time. A small-to-medium effect size was reported for both TMTB (*f*^2^ = 0.11) and reaction time (*f*^2^ = 0.11). However, it is important to point out that in looking carefully at the regression lines in both Figure 5 and Figure 6, these changes are likely being driven by 5 players who experienced roughly 1/3 of all the headers logged. As a result, these outliers are driving the reported linear relationships derived in the regression analysis. Heading exposure was not a predictor for the remaining variables.

## 4. Discussion

Youth soccer players in the United States who can start purposefully heading the ball in practices and games once they reach the age of 11 are of particular interest when it comes to studying the effects of RHI. We argue that these findings lend support to the current heading restrictions put forth back in 2015 by US Soccer and in place across the United States youth soccer playing environment. This study observed a few small improvements in neurocognitive functioning and decreases in balance/gait measures while no changes occurred in self-reported symptoms from pre- to post-season. Immediate memory, concentration, TMTA, and TMTB all had small improvements in performance over the course of the season. Performance on tandem gait single-task demonstrated a small improvement from pre- to post-season. Sway velocity and sway frequency (double leg stance) both demonstrated deficits. Changes in TMTB and reaction time were both predicted by a season of heading exposure whereby and quite intriguing a larger number of headers predicted improved times in both TMTB and reaction time.

Child SCAT5 immediate memory and concentration scores were significantly improved from pre- to post-season. Immediate memory has a maximum score of 15 and the scores reported here, 14.15 versus 14.43, are essentially the same score of 14 and demonstrate very good scores. Concentration is scored out of 6 and our pre-season score was 4.22 while the post-season score was 4.48, indicating an insignificant clinical change. The pre- and post-season scores for immediate memory and concentration are within the normative values reported for players aged 11–13 years (immediate memory: 14.1 ± 1.1 and concentration: 4.5 ± 1.1) [69] Based on the small changes in immediate memory and concentration we determined the changes to not be clinically significant as the scores essentially stayed the same. TMTA and TMTB performance both improved from pre- to post-season (24.99 s vs. 22.25 s and 57.58 s vs. 50.54 s respectively) which is likely due to a practice effect [7,26]. During the 8–10 weeks between the pre- and post-season testing, the youth at this age are rapidly growing and learning, likely leading to improved neurocognitive functioning. The results from the current study point to there being no detrimental relationship between heading exposure and neurocognitive functioning. This finding is supported by other studies in the youth, high school, and collegiate populations that examined heading exposure over one or more seasons or after an acute bout of heading with no changes in neurocognitive functioning [7,26,32,33,34,70].

Deficits in sway velocity and frequency were observed in this cohort of female youth soccer players. Cacesse et al. [8] reported similar results observing an increase in sway velocity in a population of 12–24 year old soccer players after an acute bout of heading. In another study involving an acute bout of heading, changes in postural control were recorded 24 h after the heading protocol but then returned to normal 48 h after the heading protocol [49]. In a population of collegiate soccer athletes, the high heading exposure group demonstrated worse postural control than the lower heading exposure groups suggesting that higher levels of heading exposure are related to deficits in postural control [71]. Wahlquist et al. [70] observed deficits in 3 of the 5 SWAY stances after an acute bout of heading in youth players. In contrast, there have been several studies reporting no changes in postural control following acute bouts of heading and following a season(s) of play [6,32,50,51]. Interestingly, no deficits were observed in the single foot balance measures in this cohort. It is possible that the small deficits in sway velocity and frequency involving the double-leg stance are not clinically meaningful nor related to heading exposure.

Tandem gait is a reliable concussion assessment tool that can differentiate between those who are concussed and those who are not [59]. Newer research suggests that less trials may be just as effective as the current standard [64]. Youth soccer players in the current study demonstrated a slight improvement in single-task time from pre- to post-season. A practice effect could have occurred as has been previously reported in a youth population, including high school athletes [57]. The change in single-task time, 1.13 s, is also less than the reliable change index, 5.3 s, for athletes in this age group [57]. Howell et al. [57] also reported that athletes improved on the single-task from the first to second time point. In a women’s collegiate soccer population, Caccese et al. [72] observed a small improvement in tandem gait from pre- to post-season. They also observed that those who were worse on tandem gait also had a greater exposure to higher linear acceleration head impacts [72]. This is one of the first studies to examine tandem gait pre- and post-season in youth soccer players. Our research team observed that players in the U12 and U13 age groups had difficulty completing the cognitive tasks, especially during the dual-task maneuver. We believe that the cognitive tasks may be too advanced and a better sequence of age-appropriate dual-tasks for the youth participants would be to spell a 5-letter word forward, perform addition instead of subtraction, and recite the months of the year in order. Howell et al. [57] stated that cognitive performance in the youth should be interpreted cautiously as their cognitive test performance was poor. As a reliable concussion test, tandem gait needs to be studied further in all soccer populations to determine if there are any changes due to heading exposure.

No changes in symptoms were detected in this population of female youth soccer players from pre- to post-season. This is supported by many studies involving youth through the college level soccer players and being assessed before and after a season or an acute bout of heading [9,26,70]. No detrimental relationship between heading and self-reported symptoms related to concussion has been reported [7,26]. However, soccer players have reported worse symptoms immediately after heading with the most common symptom being a headache [9,25]. We did not measure symptoms directly after heading however, there were no detected changes in symptoms between the beginning and end of the soccer season.

Heading exposure in our youth soccer cohort revealed that there is low heading exposure in games compared to practices. While generally speaking, heading exposure in games was similar across each of the three age groups, the older players (U-15 age group) had a significantly higher number of headers in practice (17) than the other two younger age groups players. Previously in collegiate populations, heading exposure for games was reported to be higher than practice heading exposure [73,74]. Kontos et al. [7] also reported higher heading exposures in games versus practices for their 13–18 year old female soccer players. However, the males in their study had more headers in practices versus games, similar to what we report. The difference in heading exposure between games and practices may be attributed to coaching style where some coaches like to focus on teaching technical skills such as passing and shooting while others like to also include heading drills to improve players’ skills in aerial challenges. Our youth cohort averaged 1.44 headers per game, less than what has been previously reported for the same age groups; including Beaudouin et al. [75] and Harris et al. [76] who both reported approximately 20 headers per game in the U12-U14 age groups. Sandmo et al. [77] observed values closer to ours at 4.4–6.4 headers per game in their U12-U14 females. When comparing our heading exposure to the other studies, we must take into consideration that they took place in Europe [75,77] and Canada [76] where restrictions on heading in youth soccer at the time were limited. It is also important to note that we did not monitor head impact magnitude as a result of each header that was logged. We certainly are aware of the fact that the magnitude of head accelerations may play a role in concussive events and not just a matter of impact exposures due to heading of a soccer ball.

There are some limitations to the current study. First, heading exposure for practices and games was only reported for those who participated in this study. There were other players who were on the same teams as our participants that did not head the ball and others that headed the ball which may have increased or decreased our heading exposure. However, these players’ heading exposures were not included in our study. Secondly, any headers that occurred outside of team games and practices were not accounted for, as some players may practice headers on their own time. Another limitation was that our cohort experienced low levels of heading exposure which may not have affected our clinical outcome measures. Changes in clinical outcome measures have been observed in older soccer populations who have higher levels of heading exposure as compared to our female youth population. Lastly, this study only included female athletes from the mid-Atlantic region of the United States and involved players from two separate clubs across two different seasons (Fall and Spring). Seasonal variations such as this may affect heading exposures. While it is current understanding that male youth players tend to head the ball more than their female aged counterparts, further study is needed to add to this belief.

## 5. Conclusions

This study examined the effects of heading exposure on self-reported symptoms, neurocognitive functioning, balance, and gait after a season in female youth soccer players. Small improvements in neurocognitive functioning and gait and slight deficits in balance were observed from pre- to post-season. All changes were not clinically relevant and likely due to a practice effect. The low heading exposure in our cohort of youth soccer players was likely not enough to elicit any changes in our studied clinical measures. In general, our clinical outcomes did not change after a season of soccer play and change scores were not predicted by heading exposure. Future study involving male youth players is suggested. Current US Soccer guidelines restricting heading exposure in youth soccer players between the ages of 11–13 should be enforced by soccer coaches and clubs as being warranted in protecting young, developing players.

## Figures and Tables

**Table 1:** Participant Demographics.

	U12	U13	U14	Total
N	19	28	14	61
Age (years)	11.1 ± 0.5	12.1 ± 0.5	12.9 ± 0.4	11.9 ± 0.8
Height (cm)	151.1 ± 7.1	154.8 ± 9.0	157.3 ± 6.6	154.2 ± 8.2
Mass (kg)	44.3 ± 7.4	48.9 ± 10.2	51.1 ± 13.2	48.0 ± 10.4

U12 = under 12 yo. U13 = under 13 yo. U14 = under 14 yo.

**Table 2:** Heading Exposure for Practices and Games.

		U12 (N = 19)	U13 (N = 28)	U14 (N = 14)	Total (N = 61)
Practices		22	26	18	66
	Headers	105	111	295	511
	Headers/Player	6.49 ± 8.67	3.49 ± 3.13	22.46 ± 13.73	10.81 ± 11.74
	Headers/Practice	4.77 ± 6.24	4.27 ± 4.51	17.16 ± 9.85	8.73 ± 8.60
Games		23	28	15	66
	Headers	11	42	25	78
	Headers/Player	0.52 ± 0.55	1.64 ± 0.90	1.90 ± 1.09	1.35 ± 0.94
	Headers/Game	0.41 ± 0.43	1.49 ± 0.25	2.41 ± 2.25	1.44 ± 1.36

U12 = under 12 yo. U13 = under 13 yo. U14 = under 14 yo. Headers/Player: the number of headers a player performed over the course of the season. Headers/Game: the number of headers a player performed per game.

**Table 3:** Outcome Measures Pre- and Post-Season.

	Pre	Post	*p* Value	Effect Size
Total Symptoms	4.34 ± 4.79	4.25 ± 4.84	0.836	
Total Symptom Severity	5.82 ± 7.75	5.79 ± 7.85	0.968	
Immediate Memory	14.15 ± 1.06	14.43 ± 0.62	0.034 *	0.32
Concentration	4.22 + 0.92	4.48 ± 1.07	0.038 *	0.27
Delayed Recall	4.13 ± 1.16	4.10 ± 1.23	0.788	
TMTA (s)	24.99 ± 7.47	22.25 ± 6.19	<0.001 *	0.39
TMTB (s)	57.58 ± 23.80	50.54 ± 17.04	0.006 *	0.32
Single-Task (s)	16.10 ± 3.90	14.97 ± 3.52	0.046 *	0.31
DTC—Word (%)	85.41 ± 46.34	75.42 ± 40.21	0.099	
DTC—Subtraction (%)	86.17 ± 58.73	83.85 ± 47.53	0.751	
DTC—Months (%)	88.61 ± 55.10	75.75 ± 37.82	0.067 *	0.27
Feet Together (%)	95.98 ± 13.60	93.34 ± 15.14	0.103	
Tandem Left (%)	79.49 ± 24.45	76.24 ± 29.68	0.471	
Tandem Right (%)	78.24 ± 27.19	79.55 ± 26.14	0.683	
Single Right (%)	55.13 ± 34.07	53.71 ± 35.50	0.781	
Single Left (%)	64.56 ± 29.71	65.19 ± 28.94	0.864	
Total Balance (%)	74.65 ± 17.62	73.89 ± 17.92	0.673	
Reaction Time (ms)	270.03 ± 48.29	277.02 ± 51.12	0.355	
SwayV—Both (cm/s)	5.10 ± 1.65	6.28 ± 3.17	0.002 *	0.44
Area—Both (cm^2^)	13.71 ± 8.91	17.55 ± 19.98	0.107	
SwayFreq—Both (Hz)	0.79 ± 0.32	0.94 ± 0.59	0.016 *	0.29
SwayV—Right (cm/s)	12.97 ± 7.36	13.32 ± 6/15	0.692	
Area—Right (cm^2^)	124.13 ± 201.42	105.12 ± 114.28	0.429	
SwayFreq—Right (Hz)	0.81 ± 0.20	0.84 ± 0.17	0.268	
SwayV—Left (cm/s)	12.20 ± 6.64	13.89 ± 12.11	0.239	
Area—Left (cm^2^)	82.56 ± 113.84	86.24 ± 94.10	0.793	
SwayFreq—Left (Hz)	0.86 ± 0.16	0.84 ± 0.17	0.296	

Pre (Season) and Post (Season): mean ± standard deviation; SwayV sway velocity; Area 95% confidence ellipse area; SwayFreq sway frequency; * denotes significance with Benjamini-Hochberg correction.

**Table 4:** Linear Regression Results for TMTB and Reaction Time.

	*R* ^2^	*B*	SE *B*	β	*p* Value
TMTB					
Headers	0.092	−0.502	0.204	−0.309	0.017
Reaction Time					
Headers	0.100	−1.582	0.619	−0.316	0.013

## Data Availability

The data presented in this study are available on request from the corresponding author due to privacy and local IRB restrictions. Please contact the corresponding author (T.W.K.) for additional steps to acquire the data.

## Supplementary Materials

The following supporting information can be downloaded at: https://www.mdpi.com/article/10.3390/sports12120342/s1, Figure S1: General health and concussion history questionnaire; Figure S2: Child scat5—symptom checklist; Figure S3: Child scat5—step 3 and 5; Figure S4: Trail making test A and B.
