# Supplementary material for: Youth Soccer Heading Exposure and Its Effects on Clinical Outcome Measures
Source: Sports (Basel). 2024 Dec 10;12(12):342. doi: 10.3390/sports12120342 (PMC11678946; doi:10.3390/sports12120342)

Supplemental Material

Figure S1

GENERAL HEALTH AND CONCUSSION HISTORY QUESTIONNAIRE

ID No.___________

UNIVERSITY OF DELAWARE – SOCCER HEADING STUDY

General Health and Concussion History Questionnaire

(All information is fully confidential and will not be shared with anyone on your team)

Section I: Demographic Information

Name ___________________________________ Year in School ________________________

Height:_________ Weight:__________ Age:_______ Gender: __________

Parent E-mail Address:___________________________________

Parent cell phone number: __________________________________

Primary Position in Soccer: (If more than one, place a 1 next to primary position, and a 2 next to secondary position)

Soccer: ☐ Forward ☐ Midfielder ☐ Defender ☐ GK

How many years have you played organized soccer (Club or School sponsored): _________

Section II: Injury History. *During the **past 6 months**, have you had an injury to any of the following body parts that caused you to miss participation for the defined time periods? Check box indicating time lost due to each injury.*

**Body Part**	**Missed Less than 7 Days**	**Missed 8-21 Days**	**Missed 22 or More Days**
Head (if concussion see below)			
Neck			
Shoulder			
Arm – Elbow			
Wrist – Hand - Fingers			
Trunk			
Hip - Thigh			
Knee			
Lower Leg – Ankle Foot - Toes			

SECTION III: History of Concussion

The following section involves answering questions related to history of previous concussions.

CONCUSSION HISTORY

Definition of Concussion: A change in brain function following a force to the head, which may be accompanied by temporary loss of consciousness, but is identified in awake individuals with measures of neurologic and cognitive dysfunction. Common concussion symptoms include:

- Headache
- Feeling slowed down
- Difficulty concentrating or focusing
- Dizziness, balance problems, loss of balance
- Fatigue/lack of energy
- Feeling in a fog
- Irritable
- Drowsiness
- Nausea
- Forgetting things (before or after the injury)
- Sensitivity to light/noise
- Blurred vision

IMPORTANT: A) A concussion can occur without being "knocked out" or unconscious B) getting your "bell rung" and "clearing the cobwebs" is a concussion

Have you ever had a concussion related to sport or other activities? ☐ Yes ☐ No If yes, how many previous concussions have you had? _______

	Sport or Non-Sport Related Concussion	The concussion was diagnosed or undiagnosed	Approximate date of Injury (mm/yyyy)	Age at time of injury	Did you lose consciousness (i.e. knocked out/blacked out)?	How long were you unconscious (seconds)?	Did/do you have difficulty remembering things before or after the injury?	How many minutes do you not remember (min)	How many days did you experience symptoms related to the injury?
Injury #1	☐Sport ☐Non-Sport	☐ Diagnosed ☐ Undiagnosed	____/____		☐Yes ☐No	________(sec) ☐ Unknown	☐Yes ☐No	________(min) ☐ Unknown	________(days) ☐ Unknown
Injury #2	☐Sport ☐Non-Sport	☐ Diagnosed ☐ Undiagnosed	____/____		☐Yes ☐No	________(sec) ☐ Unknown	☐Yes ☐No	________(min) ☐ Unknown	________(days) ☐ Unknown
Injury #3	☐Sport ☐Non-Sport	☐ Diagnosed ☐ Undiagnosed	____/____		☐Yes ☐No	________(sec) ☐ Unknown	☐Yes ☐No	________(min) ☐ Unknown	________(days) ☐ Unknown
Injury #4	☐Sport ☐Non-Sport	☐ Diagnosed ☐ Undiagnosed	____/____		☐Yes ☐No	________(sec) ☐ Unknown	☐Yes ☐No	________(min) ☐ Unknown	________(days) ☐ Unknown
Injury #5	☐Sport ☐Non-Sport	☐ Diagnosed ☐ Undiagnosed	____/____		☐Yes ☐No	________(sec) ☐ Unknown	☐Yes ☐No	________(min) ☐ Unknown	________(days) ☐ Unknown
Injury #6	☐Sport ☐Non-Sport	☐ Diagnosed ☐ Undiagnosed	____/____		☐Yes ☐No	________(sec) ☐ Unknown	☐Yes ☐No	________(min) ☐ Unknown	________(days) ☐ Unknown
Injury #7	☐Sport ☐Non-Sport	☐ Diagnosed ☐ Undiagnosed	____/____		☐Yes ☐No	________(sec) ☐ Unknown	☐Yes ☐No	________(min) ☐ Unknown	________(days) ☐ Unknown
Injury #8	☐Sport ☐Non-Sport	☐ Diagnosed ☐ Undiagnosed	____/____		☐Yes ☐No	________(sec) ☐ Unknown	☐Yes ☐No	________(min) ☐ Unknown	________(days) ☐ Unknown
Injury #9	☐Sport ☐Non-Sport	☐ Diagnosed ☐ Undiagnosed	____/____		☐Yes ☐No	________(sec) ☐ Unknown	☐Yes ☐No	________(min) ☐ Unknown	________(days) ☐ Unknown
Injury #10	☐Sport ☐Non-Sport	☐ Diagnosed ☐ Undiagnosed	____/____		☐Yes ☐No	________(sec) ☐ Unknown	☐Yes ☐No	________(min) ☐ Unknown	________(days) ☐ Unknown

Figure S2

CHILD SCAT5 – SYMPTOM CHECKLIST

2

Child Report[3]	Not at all/ Never	A little/ Rarely	Somewhat/ Sometimes	A lot/ Often
I have headaches	0	1	2	3
I feel dizzy	0	1	2	3
I feel like the room is spinning	0	1	2	3
I feel like I'm going to faint	0	1	2	3
Things are blurry when I look at them	0	1	2	3
I see double	0	1	2	3
I feel sick to my stomach	0	1	2	3
My neck hurts	0	1	2	3
I get tired a lot	0	1	2	3
I get tired easily	0	1	2	3
I have trouble paying attention	0	1	2	3
I get distracted easily	0	1	2	3
I have a hard time concentrating	0	1	2	3
I have problems remembering what people tell me	0	1	2	3
I have problems following directions	0	1	2	3
I daydream too much	0	1	2	3
I get confused	0	1	2	3
I forget things	0	1	2	3
I have problems finishing things	0	1	2	3
I have trouble figuring things out	0	1	2	3
It's hard for me to learn new things	0	1	2	3
Total number of symptoms:				of 21
Symptom severity score:				of 63
Do the symptoms get worse with physical activity?			Y	N
Do the symptoms get worse with trying to think?			Y	N

Overall rating for child to answer:

	Very bad … Very good
On a scale of 0 to 10 (where 10 is normal), how do you feel now?	0 1 2 3 4 5 6 7 8 9 10

If not 10, in what way do you feel different?:

Figure S3

CHILD SCAT5 – STEP 3 AND 5

3

STEP 3: COGNITIVE SCREENING

Standardized Assessment of Concussion - Child Version (SAC-C)[4]

Name: ______________________
DOB: ______________________
Address: ______________________
ID number: ______________________
Examiner: ______________________
Date: ______________________

IMMEDIATE MEMORY

The Immediate Memory component can be completed using the traditional 5-word per trial list or optionally using 10-words per trial to minimise any ceiling effect. All 3 trials must be administered irrespective of the number correct on the first trial. Administer at the rate of one word per second.

Please choose EITHER the 5 or 10 word list groups and circle the specific word list chosen for this test.

I am going to test your memory. I will read you a list of words and when I am done, repeat back as many words as you can remember, in any order. For Trials 2 & 3: I am going to repeat the same list again. Repeat back as many words as you can remember in any order, even if you said the word before.

List	Alternate 5 word lists					Score (of 5) Trial 1	Trial 2	Trial 3
A	Finger	Penny	Blanket	Lemon	Insect			
B	Candle	Paper	Sugar	Sandwich	Wagon			
C	Baby	Monkey	Perfume	Sunset	Iron			
D	Elbow	Apple	Carpet	Saddle	Bubble			
E	Jacket	Arrow	Pepper	Cotton	Movie			
F	Dollar	Honey	Mirror	Saddle	Anchor			
					**Immediate Memory Score**	of 15		
					**Time that last trial was completed**			

List	Alternate 10 word lists					Score (of 10) Trial 1	Trial 2	Trial 3
G	Finger<br>Candle	Penny<br>Paper	Blanket<br>Sugar	Lemon<br>Sandwich	Insect<br>Wagon			
H	Baby<br>Elbow	Monkey<br>Apple	Perfume<br>Carpet	Sunset<br>Saddle	Iron<br>Bubble			
I	Jacket<br>Dollar	Arrow<br>Honey	Pepper<br>Mirror	Cotton<br>Saddle	Movie<br>Anchor			
					**Immediate Memory Score**	of 30		
					**Time that last trial was completed**			

CONCENTRATION

DIGITS BACKWARDS

Please circle the Digit list chosen (A, B, C, D, E, F). Administer at the rate of one digit per second reading DOWN the selected column.

I am going to read a string of numbers and when I am done, you repeat them back to me in reverse order of how I read them to you. For example, if I say 7-1-9, you would say 9-1-7.

Concentration Number Lists (circle one) List A	List B	List C			
5-2	4-1	4-9	Y	N	0
4-1	9-4	6-2	Y	N	1
4-9-3	5-2-6	1-4-2	Y	N	0
6-2-9	4-1-5	6-5-8	Y	N	1
3-8-1-4	1-7-9-5	6-8-3-1	Y	N	0
3-2-7-9	4-9-6-8	3-4-8-1	Y	N	1
6-2-9-7-1	4-8-5-2-7	4-9-1-5-3	Y	N	0
1-5-2-8-6	6-1-8-4-3	6-8-2-5-1	Y	N	1
7-1-8-4-6-2	8-3-1-9-6-4	3-7-6-5-1-9	Y	N	0
5-3-9-1-4-8	7-2-4-8-5-6	9-2-6-5-1-4	Y	N	1
List D	List E	List F			
2-7	9-2	7-8	Y	N	0
5-9	6-1	5-1	Y	N	1
7-8-2	3-8-2	2-7-1	Y	N	0
9-2-6	5-1-8	4-7-9	Y	N	1
4-1-8-3	2-7-9-3	1-6-8-3	Y	N	0
9-7-2-3	2-1-6-9-	3-9-2-4	Y	N	1
1-7-9-2-6	4-1-8-6-9	2-4-7-5-8	Y	N	0
4-1-7-5-2	9-4-1-7-5	8-3-9-6-4	Y	N	1
2-6-4-8-1-7	6-9-7-3-8-2	5-8-6-2-4-9	Y	N	0
8-4-1-9-3-5	4-2-7-3-9-8	3-1-7-8-2-6	Y	N	1
		**Digits Score:**	of 5		

DAYS IN REVERSE ORDER

Now tell me the days of the week in reverse order. Start with the last day and go backward. So you'll say Sunday, Saturday. Go ahead.

Sunday - Saturday - Friday - Thursday - Wednesday - Tuesday - Monday	0 1
**Days Score**	of 1
**Concentration Total Score (Digits + Days)**	of 6

© Concussion in Sport Group 2017

Davis GA, *et al. Br J Sports Med* 2017;**0**:1–8. doi:10.1136/bjsports-2017-097492childscat5

5

STEP 5: DELAYED RECALL:

The delayed recall should be performed after 5 minutes have elapsed since the end of the Immediate Recall section. Score 1 pt. for each correct response.

Do you remember that list of words I read a few times earlier? Tell me as many words from the list as you can remember in any order.

Time Started ______

Please record each word correctly recalled. Total score equals number of words recalled.

____

____

____

Total number of words recalled accurately: of 5 or of 10

Figure S4

TRAIL MAKING TEST A AND B

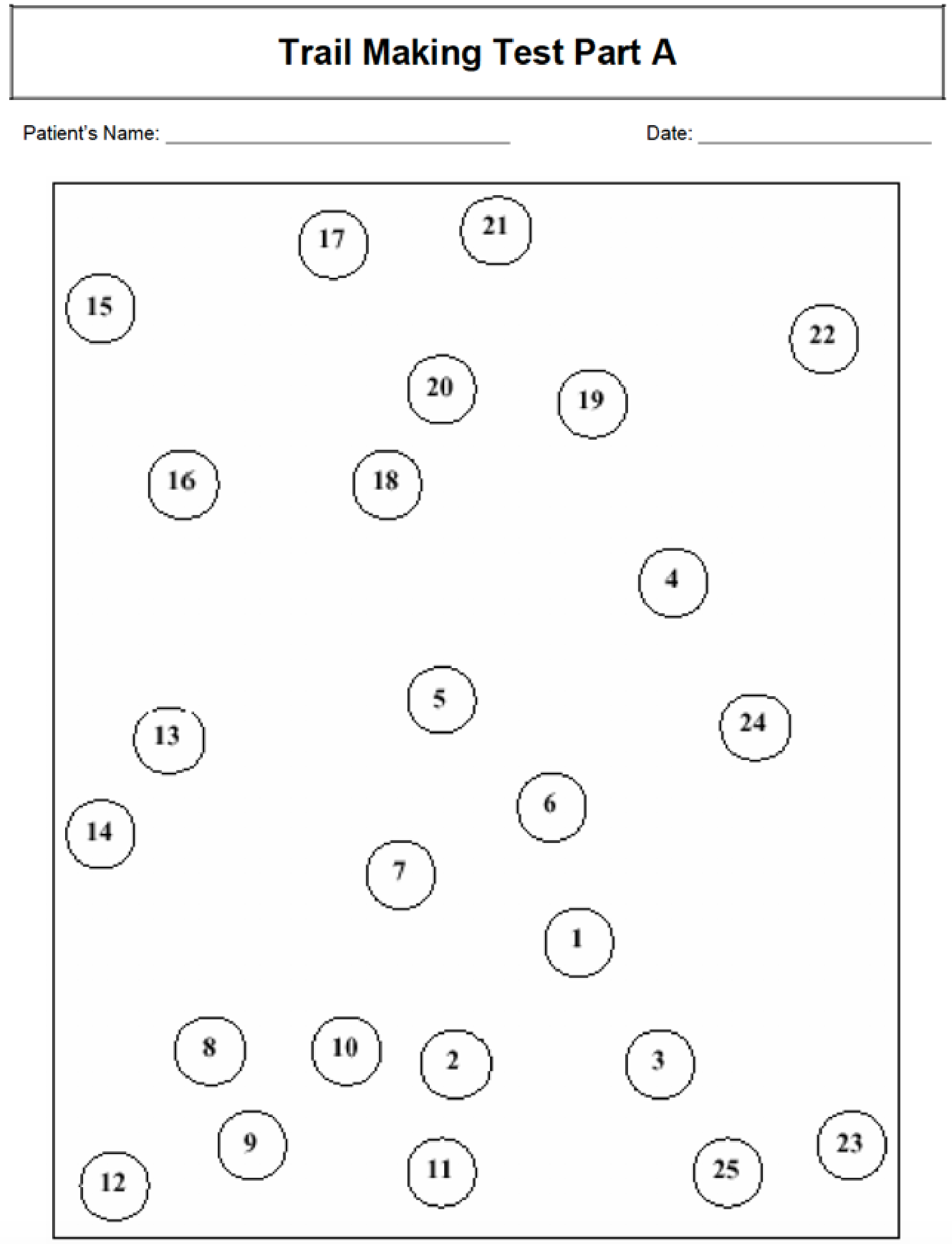

Trail Making Test Part B

Patient's Name: ______________________________ Date: ____________________

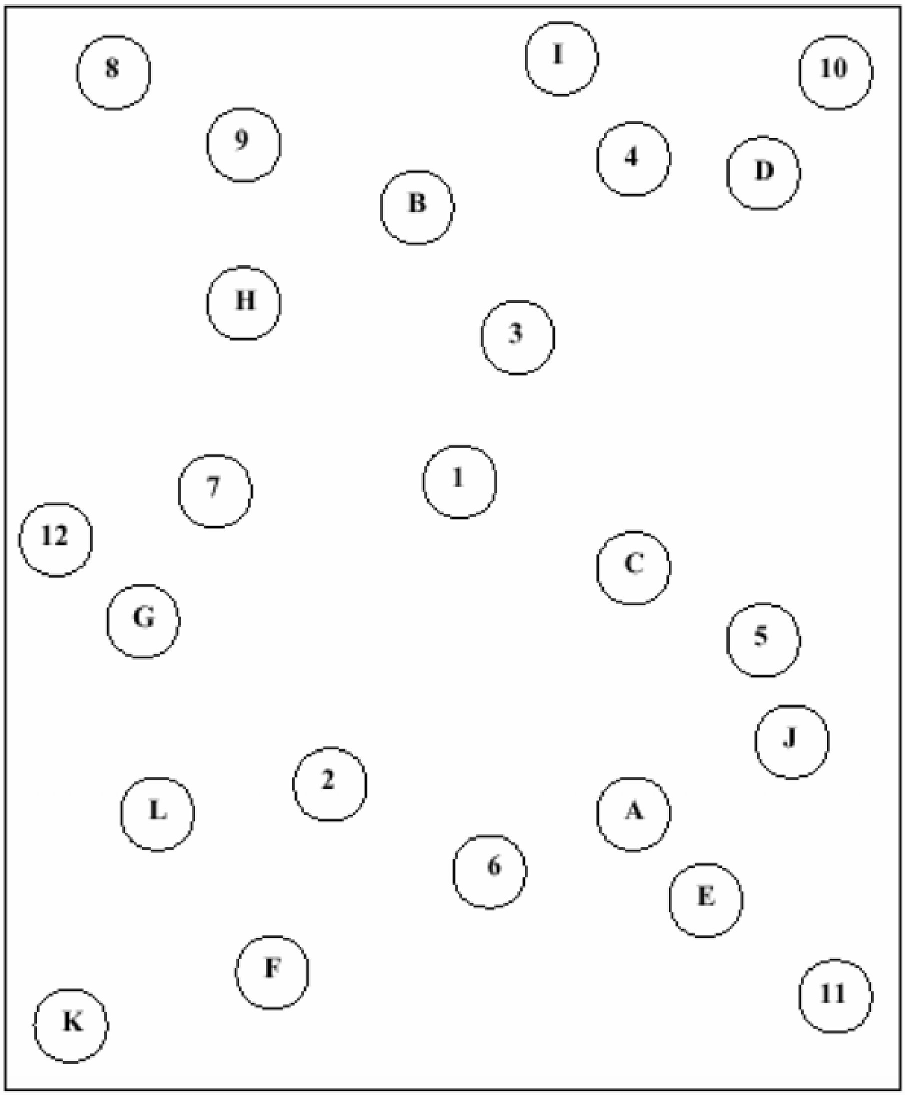

Supplement: Supplementary file 1 [file sports-12-00342-s001.zip › sports-3347528-supplementary.pdf]
